# Artificial intelligence reveals the predictions of hematological indexes in children with acute leukemia

**DOI:** 10.1186/s12885-024-12646-3

**Published:** 2024-08-12

**Authors:** Zhangkai J. Cheng, Haiyang Li, Mingtao Liu, Xing Fu, Li Liu, Zhiman Liang, Hui Gan, Baoqing Sun

**Affiliations:** 1 Department of Clinical Laboratory, National Center for Respiratory Medicine, National Clinical Research Center for Respiratory Disease, State Key Laboratory of Respiratory Disease, Guangzhou Institute of Respiratory Health, The First Affiliated Hospital of Guangzhou Medical University, Guangzhou, 510120 Guangdong China; 2 MRC Biostatistics Unit, University of Cambridge, Cambridge, CB2 0SR UK; 3 Guangzhou Laboratory, Guangzhou, 510320 China

**Keywords:** Childhood leukemia, Cancer early screening, Machine learning

## Abstract

Childhood leukemia is a prevalent form of pediatric cancer, with acute lymphoblastic leukemia (ALL) and acute myeloid leukemia (AML) being the primary manifestations. Timely treatment has significantly enhanced survival rates for children with acute leukemia. This study aimed to develop an early and comprehensive predictor for hematologic malignancies in children by analyzing nutritional biomarkers, key leukemia indicators, and granulocytes in their blood. Using a machine learning algorithm and ten indices, the blood samples of 826 children with ALL and 255 children with AML were compared to a control group of 200 healthy children. The study revealed notable differences, including higher indicators in boys compared to girls and significant variations in most biochemical indicators between leukemia patients and healthy children. Employing a random forest model resulted in an area under the curve (AUC) of 0.950 for predicting leukemia subtypes and an AUC of 0.909 for forecasting AML. This research introduces an efficient diagnostic tool for early screening of childhood blood cancers and underscores the potential of artificial intelligence in modern healthcare.

## Introduction

Leukemia represents a significant oncological challenge affecting individuals of all ages worldwide, with a marked impact on children [1]. This group of malignancies, originating from haematopoietic stem cells, disrupts normal blood cell production, compromising the immune system, and increasing susceptibility to infections [2, 3]. The aetiology of leukemia is complex, involving genetic and environmental factors, yet it remains poorly understood in many cases [4]. It is classified into various types, including acute lymphoblastic leukemia (ALL) and acute myeloid leukemia (AML), which are particularly prevalent among children, whereas other forms are more common in adults [5, 6].

Early diagnosis of leukemia is crucial for improving prognosis and survival rates. The existence of various diagnostic methods with strengths and limitations, the challenge of early leukemia diagnosis necessitates comprehensive testing, including Morphology, Immunology, Cytogenetics, and Molecular biology (MICM), to accurately identify the disease [7–9]. Morphological examination of blood and bone marrow samples under a microscope is fundamental but depends on the pathologist’s skill and may miss minimal residual disease (MRD) and early-stage leukemia [10]. Flow cytometry-based immunophenotyping identifies specific markers on leukemia cells, aiding in subtype classification, but it is time-consuming and requires sophisticated equipment [11, 12]. Cytogenetic and molecular diagnostics detect genetic abnormalities and mutations but are expensive and complex, limiting their routine use [13]. Biochemical markers like elevated lactate dehydrogenase (LDH), C-reactive protein (CRP), platelets (PLT), and hemoglobin (HB) can indicate leukemia but lack specificity and can result in false positives [14, 15]. However, The MICM diagnostic approach is complex, time-consuming, expensive, requires specialized personnel, depends heavily on sample quality, has limited sensitivity for early disease detection. Therefore, we predict the disease based on a combination of multiple indices to develop a comprehensive predictive index for leukemia in young children by integrating multiple-indices parameters with advanced machine learning techniques to enhance early detection accuracy and improve patient early treatment outcomes.

Recent efforts in leukemia research have focused on early detection and prediction, employing machine learning algorithms to analyse patient blood samples for early-stage indicators of the disease [16–19]. The search for novel biomarkers and the refinement of predictive models for leukemia diagnosis underscores the importance of this research. With the continuous advancement of treatment modalities, including chemotherapy, radiation therapy, and targeted therapy, the potential to improve patient outcomes is significant [20, 21]. However, the challenge remains in identifying reliable biomarkers for early detection, which is crucial for the timely initiation of treatment and ultimately, improving survival rates among children diagnosed with leukemia.

**Fig. 1:**
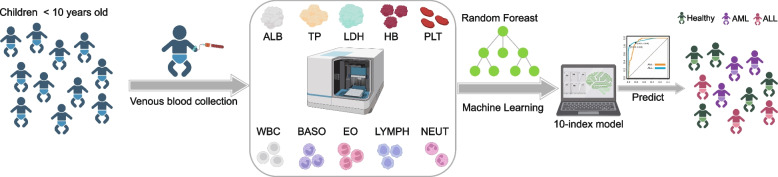
The scheme of children acute leukemia prediction model. The blood samples were collected from 1081 cases of acute leukemic children and 200 healthy children. After that, determine the protein indicators, and granulocyte ratios to train the random forest model to predict the diagnosis of acute leukemic cancer systematically

This study used the data of tests performed on leukemia patients with major subtypes (ALL, AML) in young children and healthy controls at the First Affiliated Hospital of Guangzhou Medical University between 2013 and 2023, in order to create a predictive index for leukemia. The parameters used for this purpose were albumin, total protein, total cholesterol, hemoglobin, platelets, white blood cells, neutrophil count, lymphocyte count, eosinophil count, and basophil count (see Fig. 1). Treatment for leukemia has primarily included chemotherapy, radiotherapy, and targeted therapy, and the effectiveness of treatment may depend on the subtype of leukemia, age, and the patient’s overall health. Therefore, identifying novel biomarkers for the rapid and accurate screening of leukemia will be essential to accelerate the pace of treatment development and bring the greatest benefit to patients, which is an imminent challenge.

## Materials and methods

### Participants enrollment

Our research study analyzed data from 1081 pediatric cases (<10 years old), divided into three cohorts: 826 cases of acute lymphoblastic leukemia (ALL), 255 cases of acute myeloid leukemia (AML), and 200 healthy controls. These cases were diagnosed and treated at the First Affiliated Hospital of Guangzhou Medical University, China, between July 2013 and July 2023. Healthy control subjects were randomly selected from children undergoing routine health checks at the same hospital, matched in age and sex distribution with the patient groups. All available patients diagnosed with ALL and AML during this period were included, based on comprehensive clinical and laboratory data availability. All patients utilizing a multi-parameter approach known as MICM (Morphology, Immunology, Cytogenetics, and Molecular biology) diagnosis. At our center, flow cytometry was primarily employed to analyze the surface markers on cells collected from blood or bone marrow samples to confirm the diagnosis. Specifically, cytoplasmic CD3 expression or myeloperoxidase (MPO) positivity were assessed to aid in the differentiation of leukemia subtypes. Additionally, PCR and sequencing techniques were used to detect mutations in genes such as FLT3, NPM1, and CEBPA for AML, as well as genetic alterations in genes like TEL-AML1 and MLL for ALL to double-check the diagnoses as per internationally recognized diagnostic standards. This comprehensive diagnostic approach ensured accurate classification and informed treatment strategies for patients. Clinical laboratory test results, including parameters such as albumin, total protein, total cholesterol, hemoglobin, platelets, white blood cells, neutrophil count, lymphocyte count, eosinophil count, and basophil count, were extracted from the hospital information system (HIS) and processed into Excel spreadsheets for further analysis using advanced machine learning techniques. This study was approved by the Scientific Research Project Reviews Ethics Committee of the First Affiliated Hospital of Guangzhou Medical University (Document 2021 No.K25). This study provides a comprehensive overview of the clinical indicators of ALL and AML in children, essential for advancing our understanding of the disease and its treatment.

### Key indicators screening

The selection of biomarkers (ALB, TP, LDH, HB, PLT, WBC, BASO, EO, LYMPH, and NEUT) in the leukemia prediction model was based on their clinical relevance and support from existing literature. Previous studies have demonstrated a strong correlation between the nutritional biomarkers of the human body and metabolism, and many have used ALB to evaluate tumour pathogenesis and clinical treatment [22, 23], Albumin (ALB) is a protein made by the liver and serves as a marker of nutritional status and liver function. Low levels of albumin can indicate poor nutritional status or liver dysfunction, which are common in leukemia patients [24]. Total protein (TP) measures the total amount of albumin and globulin in the blood, providing insight into the overall nutritional and health status of the patient. Altered levels of serum proteins have been observed in leukemia patients and are indicative of disease progression [25]. Lactate dehydrogenase (LDH) is an enzyme found in almost all body tissues, and elevated levels are often seen in patients with hematologic malignancies due to cell turnover and tissue breakdown. High LDH levels are commonly associated with increased tumor burden and poorer prognosis in leukemia [26, 27]. McQuilten et al. [28] pointed out in their study that Hemoglobin (HB) is an important indicator for the diagnosis of leukemia, and it has been validated. Platelets (PLT) are cell fragments involved in blood clotting, and thrombocytopenia (low platelet count) often indicates the severity of bone marrow involvement in leukemia [29]. White blood cells (WBC) are part of the immune system and fight infection, with abnormal counts being critical for the diagnosis and monitoring of leukemia [30]. Lymphocytes (LYMPH), basophils (BASO) and eosinophils (EO) are WBCs involved in the immune response, with abnormal lymphocyte counts commonly associated with leukemia [31, 32]. Neutrophils (NEUT) are the most abundant type of WBC and are crucial for fighting infections, with neutropenia indicating bone marrow suppression in leukemia [33, 34].

### Statistical analyses and visualisation

Multiple algorithms were used in data processing, such as the randomised nearest neighbour embedding (tSNE) of distribution t and the receiver operating characteristic (ROC). Furthermore, the limma toolkit (version 3.52; https://bioconductor.org/packages/limma), developed by Ritchie et al. [35], was used to perform different analyses between biochemical metrics. The data preliminary classification was visualized by the “tsne” package (version 0.1-3.1; https://cran.r-project.org/web/packages/tsne) in R studio. In addition, the ROC curve was used to evaluate and compare the effectiveness of diagnostic models and whether they are of practical value [36]. To visualize the ROC curve and AUC, we used the “pROC” package (version 1.18.0; https://cran.r-project.org/web/packages/pROC/). Data were expressed as mean SD for continuous variables and as numbers (percentages) for categorical variables, and visualized and analyzed by R studio and Python Programming. All data collection and statistical analysis of the data were performed using R version 4.2.1 and Python 3.7. The basic column bar and box plots were drawn via the “matlibplot” python package (version 3.5; https://matplotlib.org/). The subplots were visualised using the “seaborn” Python package (version 0.11.2; https://seaborn.pydata.org/). Furthermore, volcano plots were visualised using the ggplot2 package (version 3.3.6; https://cran.rproject.org/web/packages/ggplot2) in R, and a heat scatter was created using the ggplot2 and LSD package (version 2.45; https://cran.rproject.org/web/packages/LSD). To determine index differences in medical records of men and women with different cancers, we used the “beanplot” package (version 1.3.1; https://cran.rproject.org/web/packages/beanplot/). Lastly, the colour and typography optimisation was done through Adobe Illustrator (https://www.adobe.com).

### Random forest models

In this study, 10 blood indices were normalised and the data was divided into a training set (70%) and a validation set (30%) using random sampling. A random forest (RF) model was developed utilizing Python (version 3.7; http://www.Python.org) and the sklearn library (version 1.1.2; https://scikit-learn.org/stable/). The GridSearchCV module was used to optimise the parameters of the RF model, which included approximately 100 trees, each with 10 random variables and a maximum tree depth of 50, to ensure the best performance of the model. Cross-validation was utilized during the parameter tuning process to maximize the model’s stability and utility while avoiding over-fitting. Afterward, the best model was chosen based on the accuracy of the predictions made on the test set. Finally, the recognition performance was evaluated with the help of the ROC curves and the corresponding AUC values.

## Results

### Demographic characteristics of all patients

A total of 1281 participants were included in this study, 826 with acute lymphoblastic leukemia (ALL), 255 with acute myeloid leukemia (AML), and 200 healthy controls. All participants were aged<10 years and were randomly selected for comparison. The male-female ratio of participants was significantly higher in the patient groups than in the healthy controls, accounting for 62.5%, 60.8%, and 51.5% respectively.

**Fig. 2:**
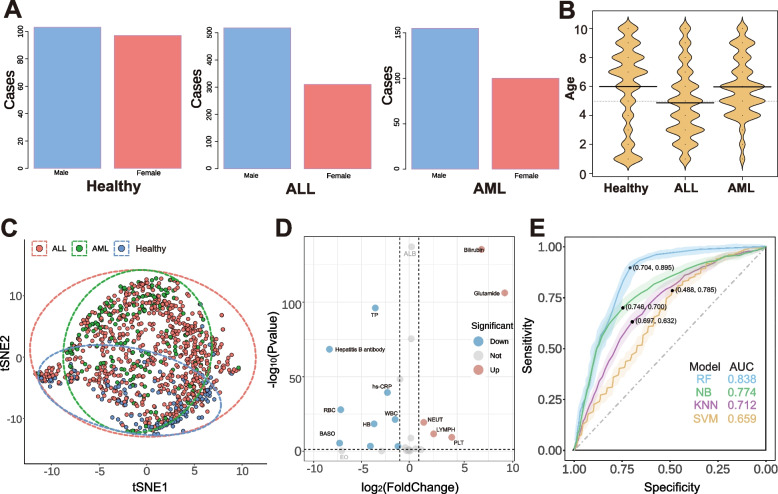
Basic information and index analysis of childhood leukemia. **A** The sex distribution of all subjects. **B** The age distribution of all participate. **C** tSNE plots of predictive indices of ALL and AML versus healthy controls. Different colored dots and dotted curves represent different cohorts and corresponding areas of concentration. **D** Volcano plot for ALL and AML versus healthy controls. **E** Comparing the performance of EO indicators under the prediction models for ALL patients: random forest (RF), naive Bayesian classification (NB), K-Nearest Neighbors(KNN:), Support Vector Machine (SVM)

The gender subgroup distribution of all participants is visualised in Fig. 2A. The difference between male and female patients was weak in the healthy control group, with no significant gender disparity in the healthy control group. The results indicate a notable gender disparity in ALL patients and AML patients, with a significantly higher proportion of boys compared to girls. This trend is consistent across both leukemia subtypes, suggesting a potential gender-related predisposition to these forms of leukemia. In contrast, the healthy control group shows a more balanced distribution between boys and girls. Figure 2B provides the age distribution of all participants. The data highlight that among ALL patients, a larger proportion are under the age of 3 compared to older age groups. This suggests that ALL is diagnosed more frequently in younger children within this cohort. In contrast, the age distribution for AML patients does not show a concentration as pronounced in this younger age range, indicating a more uniform distribution between the different age groups studied. The healthy control group displays a uniform distribution in age ranges, ensuring a broad and representative sample of the general paediatric population.

**Fig. 3:**
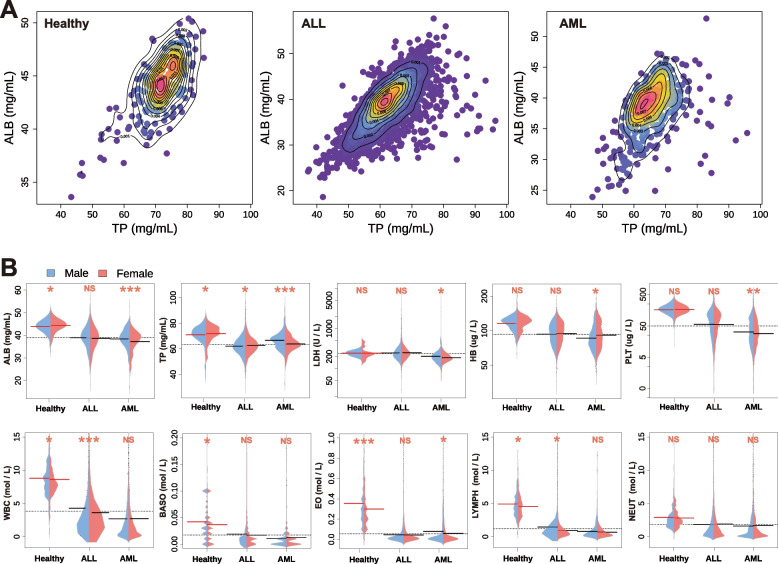
Presentation of significant indices for each cohort. **A** TP-ALB density scatters plot of healthy controls and patients with ALL and AML. **B** The distribution of 10 key indices (ALB, TP, LDH, HB, PLT, WBC, BASO, EO, LYMPH, NEUT) in investigated populations. Significance star: *** means $p < 0.001$; ** means $p < 0.01$; * means $p < 0.05$; NS means $p > 0.05$

The predictive biomarkers for the three cohorts, ALL, AML and healthy controls, were further visualised using a tSNE plot (Fig. 2C), which visualises the clustering of predictive indices for ALL and AML versus healthy controls. Different colored dots represent the three cohorts: ALL (blue), AML (orange), and healthy controls (green). The dotted curves outline the areas of concentration for each cohort. The distinct separation between these clusters indicates significant differences in the biomarker profiles of healthy children compared to those with ALL and AML, demonstrating the efficacy of these indices in differentiating between these groups. The volcano plot of the difference between ALL and AML versus healthy controls is illustrated in Fig. 2D, Points further to the right indicate indices that are significantly higher in leukemia patients compared to healthy controls, and points to the left indicate lower values. The vertical dotted lines mark the fold change threshold and the horizontal line represents the significance threshold. This graph highlights specific indices that are markedly different in leukemia patients, underscoring their potential as biomarkers for detection and diagnosis. In this study, we compared several machine learning models, including Random Forest (RF), Naive Bayes (NB), K-Nearest Neighbours (KNN), and Support Vector Machine (SVM). As shown in Fig. 2E, the RF model outperformed others across all evaluation metrics, achieving an AUC of 0.838, significantly higher than NB’s 0.774, KNN’s 0.712, and SVM’s 0.659. Additionally, the RF model demonstrated a better balance with sensitivity of 0.895 and specificity of 0.704, making it a more robust choice for the classification task. Based on these results, we selected the RF model for further analysis and application.

### Difference significance analysis of indices among ALL, AML, and healthy controls

To further investigate this correlation, in this study, we analysed 10 potential indices in detail and plotted the scatter density map of TP against ALB (Fig. 3A). The data points were separated by contour lines of different colours, allowing us to visually assess the density distribution between the scattered points. The visual distribution plots revealed that healthy controls had a scattered distribution, with ALB concentrated around 45 mg/mL and TP around 70 mg/mL, while ALL patients were centralised around 40 mg/mL and 60 mg/mL for ALB and TP, respectively. In contrast, the distribution of ALB and TP in patients with AML was relatively dispersed and the contents were rather low. In general, there were significant differences between each cohort.

**Fig. 4:**
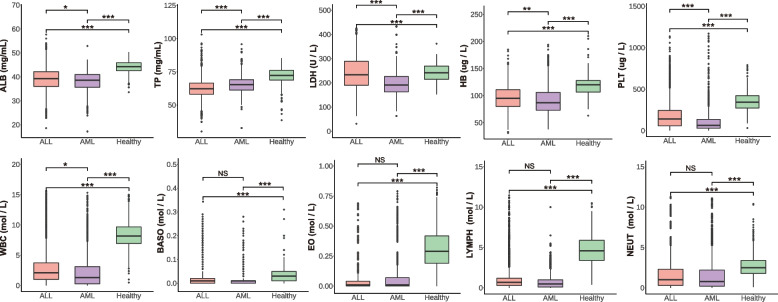
Comparative significance of ten key indices among ALL, AML, and healthy controls. Significance star: *** means $p < 0.001$; ** means $P < 0.01$; * means $p < 0.05$; NS means $p > 0.05$

To visualise gender differences, we selected 10 potential indices as our study objects and integrated the 10 indices of all participants in this study. Figure 3B shows the distribution and statistical significance of 10 potentially valuable indices in this analysis between genders among the three cohorts. We found that ALL and AML had very significant differences compared to the healthy population. Additionally, in ALL patients, there was a significant difference in WBC between genders, while in AML patients, ALB, TP, and PLT showed significant differences between genders in the analysis of each cohort. Besides, the above 10 indices were also drawn in a box chart (Fig. 4), the results highlighted significant differences between patients and healthy controls, as well as statistically significant differences in TP and LDH in ALL and AML. However, HB, ALB, and WBC showed only a few differences in this comparison. However, BASO, EO, LYMPH and NEUT were not significantly different in patients with ALL and AML.

### Detailed correlational analysis in key indices in three cohorts

**Fig. 5:**
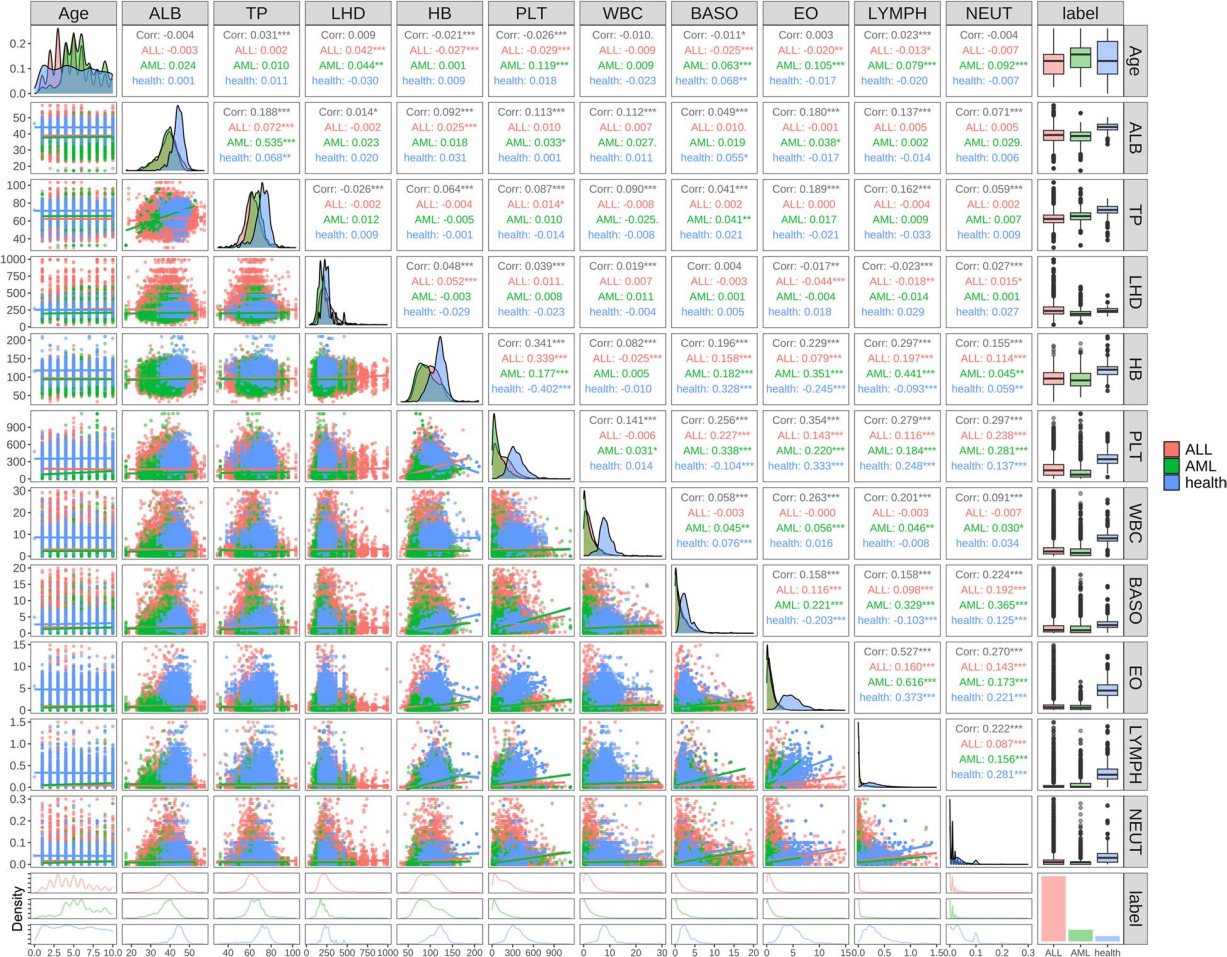
The correlation analysis between ALL, AML patients, and healthy controls in potential variables. Potential valuable variables were included in pairwise parameter combination analysis to visualize the distribution differences between patients with ALL and AML and healthy controls

Many studies have demonstrated the indices link between ALL, AML, and human metabolism [37]. In our correlation analysis, we used paired analysis to compare the distribution of 10 indices in each cohort. The Fig. 5 reveals several notable correlations in the blood parameters of ALL(red), AML(green), and healthy individuals (blue). In AML patients, a strong positive correlation (0.341***) between HD and PLT indicates that higher HD levels are associated with increased platelet counts, but in the healthy population exhibit a significant negative correlation (-0.402***), suggesting that as hemoglobin levels decrease, platelet counts tend to increase. Additionally, there is a striking positive correlation (0.527***) between EO and LYMPH. These specific data values highlight significant hematological alterations in leukemia patients compared to healthy individuals. Most of the content levels of each index in the healthy control were covered by ALL and AML patients. The lower left part of the result represented a dispersed distribution of focused indices, while the upper right part showed a concentrated distribution of centralised indices and the diagonal part illustrated the density distribution of anchor indices. We observed that the ALB, TP, HB, and PLT of healthy controls were mostly distributed above ALL and AML patients. In contrast, WBC, BASO and NEUT of ALL and AML patients were distributed covered healthy distribution. Notably, ALB and TP demonstrated the best correlation among the distribution of 10 indices and presented a well-linear distribution.

In addition, combining EO with other indices allowed healthy controls to be separated mainly from ALL and AML patients. Similarly, correlation analysis among all indices revealed that the distribution of TP and ALB, as well as of PLT, WBC, BASO, EO and LYMPH, showed a similar trend. Specifically, the former presented an elliptic triangle, while the latter took the shape of a fan-shaped inverted triangle.

**Fig. 6:**
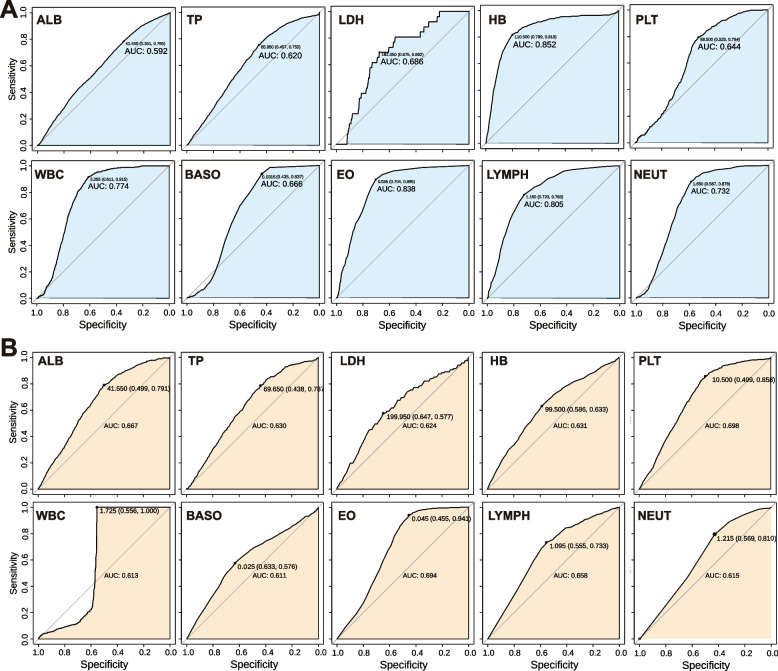
Display the predictive effects of the ROC curve of 10 individual indices in ALL (**A**), AML (**B**)

### Relevant exploration of ALL and AML in RF model

**Fig. 7:**
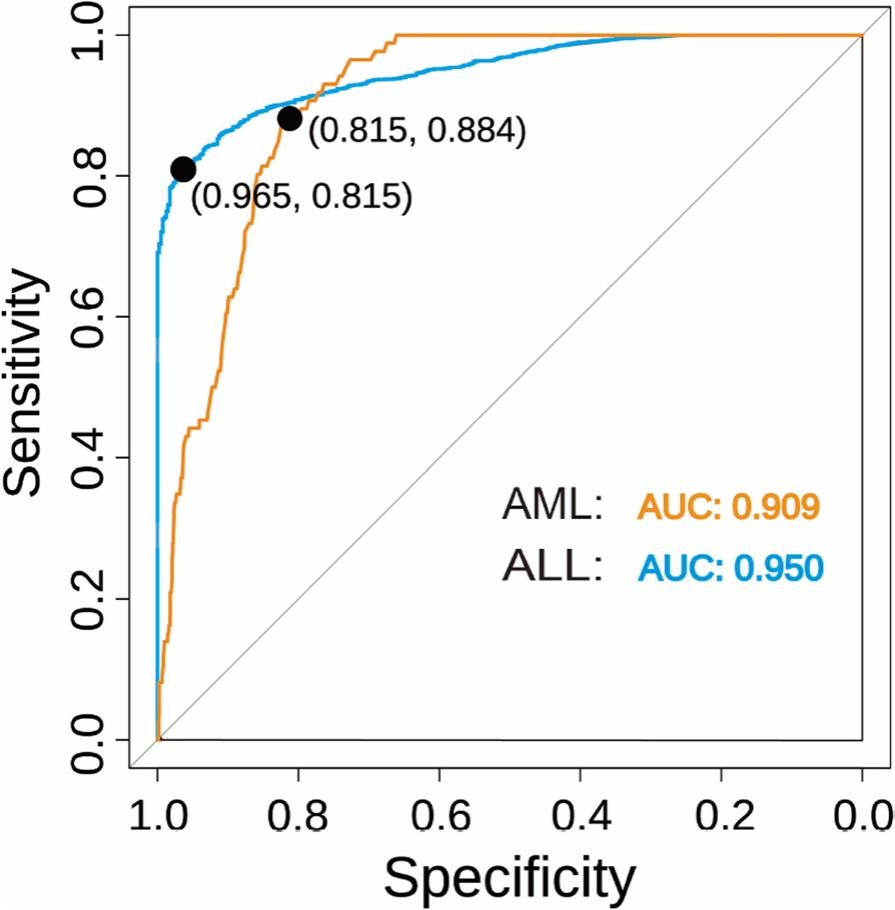
The ROC performance of the RF prediction model combining 10 indices for ALL and AML was demonstrated

After RF model prediction, for healthy controls and ALL, we imported patients with AML into the classifier for comparative analysis, where the ROC curve and the AUC of the biomarkers focused above were calculated. Therefore, as shown in Fig. 6A and B, we performed an exploration of the ROC single index prediction model for the 10 key indices to observe the efficacy of this model and further study the validity of the indices in patients with ALL and AML. In the ROC analysis of 10 markers selected in ALL patients, ALB surprisingly had a poor predictive effect. The AUCs of the other 9 indices in ALL were greater than 0.6, while HB and EO showed a better predictive effect of more than 0.8. The AUC of PLT and EO, which may produce a stronger predictive effect, was less than 0.7 in the study of patients with AML, although the AUC of all markers was greater than 0.6. In ALL, the sensitivity of the curve is mostly greater than 1, and the sensitivity of leukocytes and granulocytes is above 0.9, but the specificity of the prediction of the model is relatively poor. In general, the results indicate that EO has a superior predictive effect in patients with ALL and AML. The AUC of this model was confirmed to be 0.950 and 0.909 in patients with ALL and AML, respectively, when combined with the 10 key indices emphasized in this research (Fig. 7).

The ALL prediction model’s AUC achieved 0.950 in Fig. 7, while the sensitivity and specificity were 0.965 and 0.815, respectively. However, the AML prediction model was a little worse in ALL aspects, with an AUC of 0.909, but slightly higher specificity than the ALL value of 0.884.

## Discussion

In our study, we conducted a detailed statistical analysis of clinical laboratory test data from 1081 leukemia patients, 826 with ALL and 255 with AML, collected over a 10-year period. Data were used to identify changes in biomarkers and applied to the Random Forest (RF) model to develop a diagnostic and prognostic prediction model for ALL and AML. This approach demonstrated high accuracy in prediction. In recent years, childhood malignancies, including acute leukemias, have emerged as a significant source of morbidity and mortality among children under the age of 10 [38]. To effectively reduce the prevalence of this widespread disease, early detection and prediction of childhood leukemia are of the utmost importance. The international incidence of leukemia is 7.6 per 100,000 people for men and 5.4 per 100,000 people for women [39]. Our study reflects these ratios and highlights the distribution of indices for healthy individuals and leukemia patients. The volcano plot revealed that the leukemia patients’ indices are primarily in the region of up and down regulation, with a significant fold change in some indices, demonstrating the distinction between leukemia patients and healthy individuals.

Nutrition is an important factor in the prognosis of cancer patients. Serum albumin (ALB) is a good indicator of nutritional health and has been used as a predictor of leukemia Katsuya et al. [40] to investigate its prognostic effect. Healthy children in China have serotonin levels in the range of 40-55 mg/mL and total serum protein levels in the range of 65-84 mg/mL [41], and Total Protein <60 g/L or Albumin <25 g/L is referred to as hypoproteinemia [42]. Our study found that leukemia patients had more hypoproteinemia than healthy children, and the TP and ALB indexes of children with two kinds of leukemia are lower than those of healthy children. As shown in Fig. 3, most leukemia patients have low levels of albumin, in connection with a report by Wang et al. [43] demonstrating that leukemias are associated with lower albumin . Also confirmed by Sadek et al. [44] in a study found that patients with acute myeloid leukemia (AML) had significantly lower levels of albumin compared to healthy individuals. Among these low levels of albumin, patients were more likely to experience treatment-related toxicities, such as infections, compared to patients with normal ones [45, 46].

In this study, the random forest model demonstrated excellent performance, with an AUC of 0.950 for the ALL prediction model and an AUC of 0.909 for the AML prediction model. These results surpass those of previous studies. For instance, Mishra et al. [16] utilized a Gray Level Co-occurrence Matrix (GLCM) method to analyze cell morphology, achieving an AUC of 0.87. Similarly, Das et al. [19] employed support vector machines (SVM) to improve ALL detection and compared the effectiveness of different machine learning classifiers. Researchers have also analyzed patient BMI [47], gut microbiota [17], and daily diet [48] to screen for risk indices. Additionally, metabolic imbalance biomarkers in the exploratory stage, such as changes in glucose [14], might be important diagnostic tools for detecting leukemias, particularly in differentiating between different types of leukemias. Furthermore, key markers of leukemia, including Hemoglobin (HB), White Blood Cell Count (WBC), and Platelet Count (PLT), were identified [49, 50]. The results showed significant differences between healthy and leukemic children, as well as considerable differences between the subtypes ALL and AML (Fig. 4). These three indicators were particularly focused on in some cases. Bain [51] stated that hemoglobin levels could be an important diagnostic tool in detecting leukemia, with low levels indicating the presence of the disease and anemia often being one of the first symptoms. Regarding the white blood cell count, Kiem Hao et al. [52] noted that it could be more than three times higher in leukemia patients than in healthy individuals. This increase can lead to a decrease in the number of red blood cells and platelets, resulting in anemia and a greater risk of bleeding and infections. Platelet count is another crucial parameter in the diagnosis of leukemia. Surprisingly, a decrease in platelet count, combined with an increase in white blood cell count, can strongly indicate leukemia [53]. In some cases, the platelet count may be the only abnormal finding leading to a diagnosis of leukemia [54].

The analysis of Fig. 5 highlights several important correlations among blood parameters in ALL, AML, and healthy individuals. In AML patients, there is a strong positive correlation (0.341***) between hemoglobin (HB) and platelet count (PLT), indicating that higher hemoglobin levels are associated with increased platelet counts. Conversely, in healthy individuals, a significant negative correlation (-0.402***) suggests that as hemoglobin levels decrease, platelet counts tend to increase. This contrast underscores the distinct hematological profiles between leukemia patients and healthy individuals. Additionally, a notable positive correlation (0.527***) between eosinophils (EO) and lymphocytes (LYMPH) in leukemia patients suggests a linked alteration in the immune response. The distribution patterns further reveal that healthy controls generally have higher levels of albumin (ALB), total protein (TP), hemoglobin (HB), and platelet count (PLT) compared to ALL and AML patients. In contrast, white blood cells (WBC), basophils (BASO), and neutrophils (NEUT) levels in ALL and AML patients cover the distribution range seen in healthy individuals, indicating elevated levels in leukemia patients. These findings emphasize the significant hematological changes in leukemia patients compared to healthy individuals and underscore the importance of integrating multiple hematological indices for improved diagnostic accuracy. Understanding these correlations provides valuable insights into the disease’s pathophysiology and aids in developing targeted diagnostic and therapeutic strategies.

The results of the study could have important implications for the early detection and treatment of these types of leukemia, especially in children where these malignancies are a significant source of morbidity and mortality. In clinical practice, our model performance could be used to screening and diagnostic tests for ALL and AML, which could lead to earlier detection and more effective treatment (Fig. 7). The model could also be used to monitor the progression of the disease and evaluate the effectiveness of treatment. However, further validation of the model in a clinical setting is necessary before it can be widely adopted. Future research can build upon these findings by further refining the predictive model and incorporating other factors that can affect the prognosis of patients with leukemia, such as environmental and lifestyle factors, to increase the precision of the model. Additionally, larger and more diverse patient populations can be studied to validate the findings of the current study and to make the model more generalizable.

## Conclusion

In this study, we aimed to investigate the differences in common blood indices between healthy children and those with acute leukemia, as well as to analyze the relationship between these indices and the gender of children. To achieve this goal, we used a 10-key index RF model for the prediction of leukemia subtypes. The results indicated that the proposed method achieved impressive predictive performance, with an AUC of 0.950 for AML and 0.909 for ALL. Therefore, these findings demonstrate that the proposed model needs to be validated prospectively as an auxiliary tool for the detection of blood cancer.

## Data Availability

The original coding contributions presented in this study are included in DOI:10.17632/fz7d6x4bwx.1, further inquiries can be directed to the corresponding author.
